# Antibody-Free Mass Spectrometry Identification of Vascular Integrity Markers in Major Trauma

**DOI:** 10.1089/neur.2021.0007

**Published:** 2021-07-01

**Authors:** H.E. Hinson, Jon Jacobs, Shannon McWeeney, Ann Wachana, Tujin Shi, Kendall Martin, Karin Rodland

**Affiliations:** ^1^ Departments of Neurology, Developmental and Cancer Biology, Oregon Health & Sciences University, Portland, Oregon, USA.; ^2^ Environmental and Molecular Sciences Laboratory, Pacific Northwest National Laboratories, Richland, Washington, USA.; ^3^ Division of Bioinformatics and Computational Biology, Department of Medical Informatics and Clinical Epidemiology, Developmental and Cancer Biology, Oregon Health & Sciences University, Portland, Oregon, USA.; ^4^ Portland State University, Portland, Oregon, USA.; ^5^ Department of Cell, Developmental and Cancer Biology, Oregon Health & Sciences University, Portland, Oregon, USA.

**Keywords:** biomarkers, blood–brain barrier dysfunction, endothelial activation, mass spectrometry, proteomics

## Abstract

Antibody mediated strategies for protein biomarker detection are common, but may limit discovery. We hypothesized that the use of antibody-free proteomics is feasible for detecting protein biomarkers in plasma of patients sustaining major trauma. A subset of subjects with major trauma from a prospective observational trial were analyzed. Patients were assigned to one of four groups based on their presenting Abbreviated Injury Severity Score (AIS). Sensitive, antibody-free selective reaction monitoring (SRM) mass spectrometry (MS), with spiked-in isotopically labeled synthetic peptides, was used for targeted protein quantification of a panel of 10 prospective targets. An overall tiered sensitivity analytical approach was used for peptide detection and quantification based upon plasma immunoaffinity depletion and PRISM fractionation. Forty-four patients were included in the analysis, of which 82% were men with a mean age of 50 (±19) years. Half had isolated head injury (*n* = 22), with the remaining patients experiencing multiple injuries or polytrauma (*n* = 14), isolated body injury (*n* = 2), or minor injury (*n* = 6). Peptides from 3 proteins (vascular adhesion molecule 1 [VCAM1], intercellular adhesion molecule 1 [ICAM1], and matrix metalloproteinase 9 [MMP9]) were detected and quantified in non-depleted processed plasma. Peptides from 2 proteins (angiopoietin 2 [Ang2] and plasminogen activator inhibitor-1 [PAI1]) were detected and quantification in depleted plasma, whereas the remaining 5 of the 10 prospective targets were undetected. VCAM1 (*p* = 0.02) and MMP9 (*p* = 0.03) were significantly upregulated in in the major trauma groups (1–3) versus mild injury group (4), whereas the others were not. There were no differences in protein expression between patients with traumatic brain injury (TBI; groups 1 and 2) versus those without TBI (groups 3 and 4). We detected non-specific upregulation of proteins reflecting blood–brain barrier breakdown in severely injured patients, indicating label-free MS techniques are feasible and may be informative.

## Introduction

Traumatic brain injury (TBI) is a complex, multi-faceted disease process encompassing numerous pathophysiological mechanisms. To harness this complexity, clinicians and investigators have actively pursued fluid-based biomarkers to assist with stratification, monitoring, and prognostication after TBI, with some modest successes. For example, the U.S. Food and Drug Administration's recent approval of a blood test combining ubiquitin C-terminal hydrolase-L1 and glial fibrillary acidic protein to assist decision-making regarding the need for head computed tomography (CT) after mild TBI.^1^ However, there remains a substantial gap to fill by identifying and validating new blood-based, protein biomarkers.

Recent proteomic investigations suggest that there is a tremendous gulf between known and potential biomarkers, and that prior research has not necessarily focused on the highest-yield candidates representing neurological injury.^2^ With the advent of robust high-throughput omics techniques, we are now able to interrogate the human proteome in ways not previously feasible. Mass spectrometry (MS) has the ability to detect, identify, and quantify proteins in a biological sample through the isolation and measurement of ion masses. In a bottom-up approach, proteins are broken down into their specific amino-acid-containing peptide sequences, which are then charged and accelerated so that their masses might be detected and quantified. Proteins are then identified by mapping the constituent peptide mass signature. This can be performed globally, utilizing a species-specific protein database to facilitate identification of the detected proteins via tandem MS fragmentation, or targeted, where known peptide/protein targets are searched for in the MS analysis, often after the inclusion of sequence-specific heavy labeled standard peptides. Although MS has been used for protein detection for decades, technical advances in sensitivity and resolution have made its use in biomarker discovery and quantification increasingly feasible.^3,4^ Still, application to TBI has been limited thus far, due to the lack of adoption of MS technology into the field, the technical expertise required, and skills needed for data interpretation.

There are a number of unknowns in applying MS approaches for protein detection in moderate-to-severe TBI plasma samples. One concern involves plasma from severely injured patients, especially those with multiple extracranial injuries, where the plasma would likely contain a milieu of protein components increasing the “noise” in providing adequate signal for detection. How much processing, such as the depletion of abundant proteins such as albumin, might be needed to obtain reliable signal? Depletion, or removal of high-abundance plasma proteins via affinity chromatography, decreases the dynamic range of the overall protein abundance in plasma and improves the ability to detect lower-abundance proteins, which are often the more informative ones. The objective of this pilot investigation was to investigate the feasibility of detection and quantification of known protein biomarkers in the plasma utilizing a targeted MS-based platform, focusing on a broad swath of critically ill trauma patients with a variety of injury types and severities.

## Methods

### Participants and plasma samples

We examined a subset of subjects from the Fever And Inflammation in Neurotrauma (FAINT) cohort, selected to represent a broad range of ages and injury types and injury severity for this pilot investigation. Details regarding patient identification and enrollment have been published previously,^5^ but in brief, subjects were prospectively enrolled if they experienced trauma severe enough to warrant intensive care unit (ICU) admission and informed consent was obtained from the subject or their legally authorized representative. Retrospectively after discharge, patients were assigned to one of four groups based on their presenting Abbreviated Injury Severity Score (AIS):

1. Multiple injuries or polytrauma: Head AIS score >2, one other region >2
2. Isolated head: Head AIS score >2, all other regions <3
3. Isolated body: One region >2, excluding head/face
4. Minor injury: No region with AIS >2

Baseline blood samples were collected within 6 h of the antecedent trauma. Plasma samples were obtained on admission (<8 h from the trauma), and banked at −80°C for subsequent analysis. Institutional review board approval of the protocol was obtained prior to study commencement.

### Protein detection with mass spectrometry (MS)

MS analysis was performed using a nanoACQUITY UPLC^®^ system (Waters Cooperation, Milford, MA, USA) coupled online to a TSQ Vantage triple quadrupole mass spectrometer (Thermo Scientific, San Jose, CA, USA). The liquid chromatography (LC) selective reaction monitoring (SRM) platform was configured and utilized as previously described.^6^ SRM assays were developed using spiked-in isotopically labeled synthetic peptides for targeted protein quantification of a panel of 10 prospective targets: angiopoietin 1 and 2 (Ang1, Ang2), monocyte chemoattractant protein 1 (MCP1*), tumor necrosis factor alpha (TNFA*), vascular adhesion molecule 1 (VCAM1*), intercellular adhesion molecule 1 (ICAM1*), vascular endothelial growth factor D (VEGFD*), transforming growth factor beta-1 (TGFB1*), plasminogen activator inhibitor-1 (PAI1*), and matrix metalloproteinase 9 (MMP9). The majority (*7/10) of these markers had previously been detected in separate aliquots of these patient samples as part of a prior investigation using a Luminex human analyte platform that screens secreted proteins using multi-plex fluorescent immunoassay (data not shown).

Multiple peptides for each target were identified and optimized for precursor-to-fragment ion transitions using the SRM platform (see Supplementary Table S1 for peptide sequences). An overall tiered sensitivity analytical approach was used for peptide detection and quantification based upon plasma immunoaffinity depletion of 14 specific highly abundant proteins using a ProteomeLabTM 12.7 × 79.0-mm human IgY14 LC10 affinity LC column (Beckman Coulter, Fullerton, CA, USA). Values for detected proteins were measured in comparison to a unitless reference standard; comparisons made via ratios (detected protein/reference standard = ratio), as opposed to plasma concentrations.

### Statistical analysis

Descriptive statistics (mean and standard deviation [SD] or median and interquartile range [IQR] for continuous variables; frequencies and percentages for categorical variables) were used to describe clinical data by group. Pearson's correlation coefficients (cc) were calculated to assess correlation between markers, and confidence intervals (CIs) reported. Protein data were highly skewed, and were log transformed prior to analysis. After transformation, parametric continuous data were analyzed with *t* tests. Significance was set at *p* ≤ 0.05. All analyses were conducted in R. (R Core Team (2019). R: A language and environment for statistical computing. R Foundation for Statistical Computing, Vienna, Austria. https://www.R-project.org/)

## Results

### Participants represented a broad swath of acute trauma

A representative sample of 44 patients (MS cohort) was selected from the FAINT cohort (*n* = 268) for analysis. Demographics and injury severity are listed for the overall FAINT cohort and for the MS cohort in Table 1, including injury type subgroups. The majority of subjects either had isolated head injury or multiple injuries (*n* = 36), with the remaining subjects experiencing isolated body injury (*n* = 2) or mild injury (*n* = 6). Injury severity and Glasgow Coma Scale (GCS) score varied depending on injury subtype, with those with multiple injures having the highest median injury severity score (ISS) and lowest median admission GCS score. Few presented with shock, except those with multiple injuries, based on base deficit abnormalities. Blood samples were obtained very shortly after the antecedent trauma (2 [±1] h) overall.

**Table 1.** Demographics and Injury Severity of the FAINT Cohort, Overall Cohort, and Subgroups

	All FAINT	MS cohort	Isolated head	Multiple	Isolated body	Minor injury
Male sex (%)	72%	82%	82%	79%	100%	83%
Age (± SD), years	54 (± 20)	50 (± 19)	56 (± 19)	47 (± 21)	39 (± 26)	41 (± 15)
ISS [IQR]	19 [14-26]	21 [16-31]	19 [17-25]	35 [30-45]	19 [17-22]	3 [1-5]
GCS [IQR]	15 [14-15]	14 [5-15]	14 [4-14]	14 [4-14]	15 [14-15]	15 [15-15]
Time since trauma (± SD), h	3 (± 2)	2 (± 1)	2 (± 1)	2 (± 1)	1 (± 1)	4 (± 2)
Base deficit	2 (± 3)	2 (± 3)	1 (± 3)	3 (± 3)	0	1 (± 1)

Despite the limited sampling, the MS cohort was representative of the overall cohort. FAINT, Fever And Inflammation in Neurotrauma; GCS, Glasgow Coma Scale; IQR, interquartile range; ISS, injury severity score; MS, mass spectrometry; SD, standard deviation.

### Detection of proteins of interest

Peptides from three proteins (VCAM1, ICAM1, and MMP9) were detected and quantified in non-depleted processed plasma. Peptides from two proteins (Ang2 and PAI1) were detected and quantified after immunoaffinity depletion of the top 14 plasma proteins (see Methods section for details). The remaining targets were not identified.

**FIG. 1.**
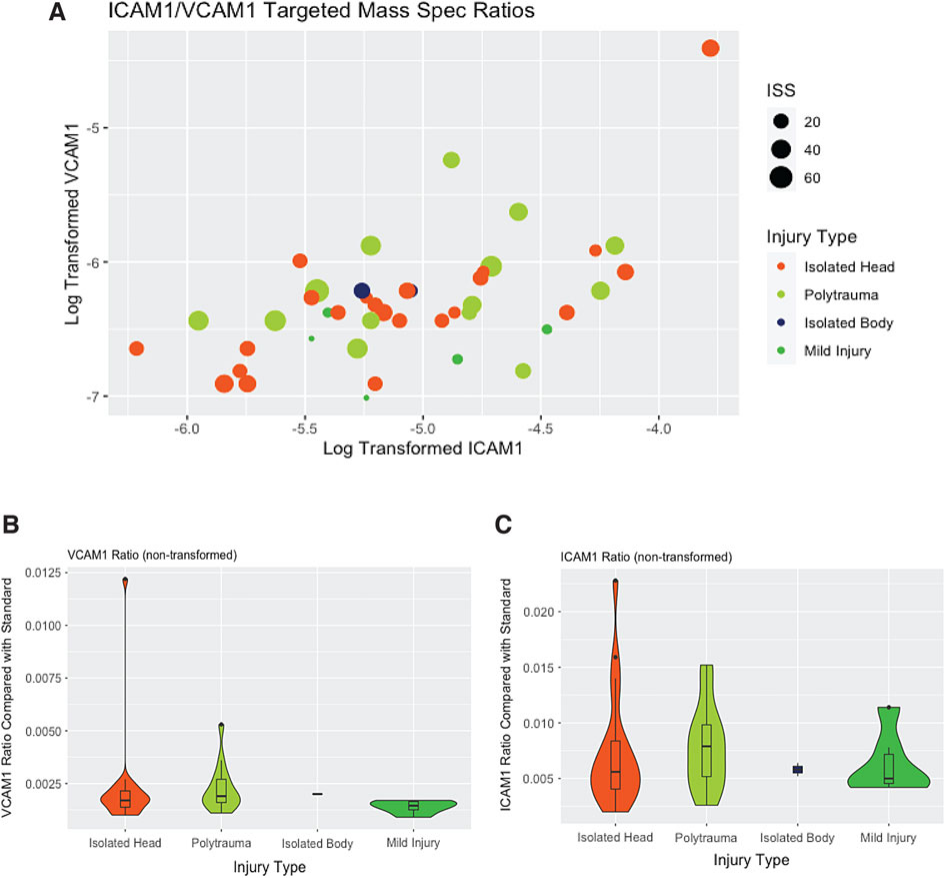
**(A)** Logarithmic transformations of VCAM1 and ICAM1 plotted against one another, with trauma type and ISS. VCAM1 and ICAM1 are significantly, moderately correlated (cc = 0.56, 95%CI: 0.32-0.74, *p* < 0.001). Violin plots of the non-transformed, raw distributions of the ratios of VCAM1 **(B)** and ICAM1 **(C)**, respectively. cc, Pearson's correlation coefficient; CI, confidence interval; ICAM1, intercellular adhesion molecule 1; ISS, injury severity score; VCAM1, vascular adhesion molecule 1.

**FIG. 2.**
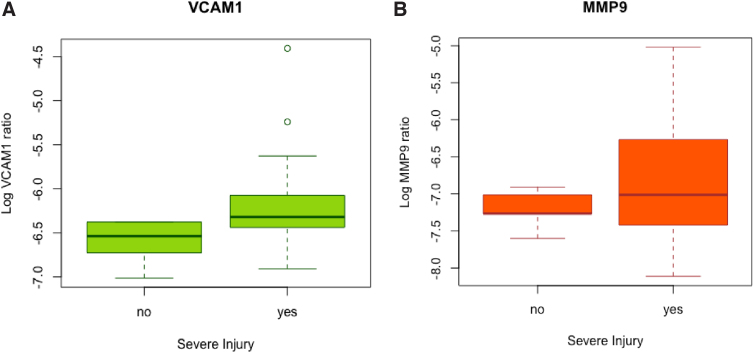
Logarithmic transformations of VCAM1 **(A)** and MMP9 **(B)** by injury severity. Levels of VCAM1 and MMP9 were higher in patients with severe injury (isolated head injury + multiple injuries + isolated body injury) versus minor injury (*p* = 0.02 and *p* = 0.03, respectively). MMP9, matrix metalloproteinase 9; VCAM1, vascular adhesion molecule 1.

**FIG. 3.**
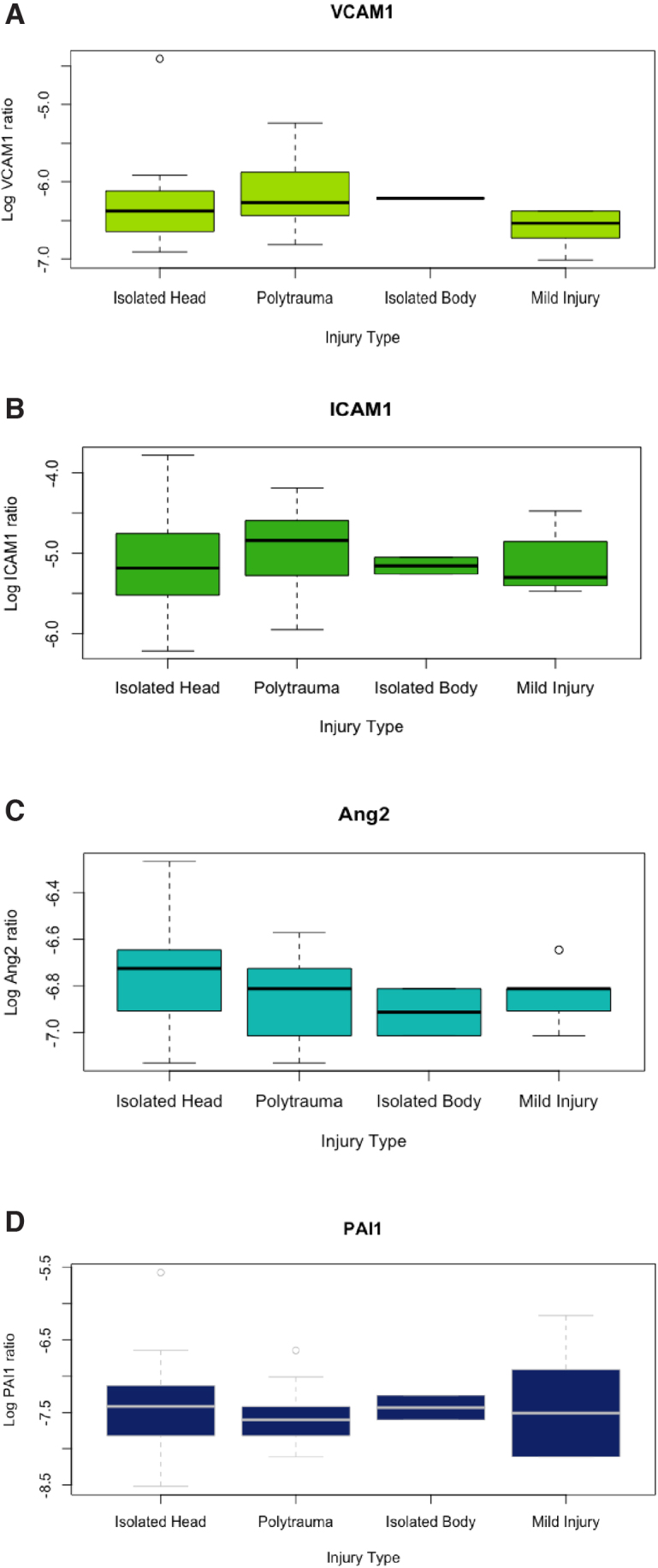
Logarithmic transformations of VCAM1 **(A)**, ICAM1 **(B)**, Ang2 **(C)**, and PAI1 **(D)** by injury type. There were no statistically significant differences by injury type in any of the detected proteins. Ang2, angiopoietin 2; PAI1, plasminogen activator inhibitor-1; ICAM1, intercellular adhesion molecule 1; VCAM1, vascular adhesion molecule 1.

Figure 1 depicts logarithmic transformations of VCAM1 and ICAM1 plotted against one another, with trauma type and ISS. VCAM1 and ICAM1 are moderately positively correlated (cc = 0.56, 95% CI: 0.32-0.74, *p* < 0.001). Non-transformed, raw values of VCAM1 and ICAM1 are also shown in Figure 1 to demonstrate the distributions. No other detected markers were significantly correlated with each other, or with the covariates of age, ISS, or admission GCS score, with one exception. MMP9 ratios were weakly, positively correlated with ISS (cc = 0.44, 95% CI: 0.17-0.65, *p* = 0.003). When patients with severe injury (multiple injuries + isolated head injury + isolated body injury) were compared with those with minor injury, those with severe injury had significantly higher levels of VCAM1 (*p* = 0.02) and MMP9 (*p* = 0.03) (Fig. 2). Levels of ICAM1, PAI1, and ANG2 were similar. In patients grouped by presence of TBI (multiple injuries + isolated head injury) versus those without TBI (isolated body injury + minor injury), no differences in any of the markers were observed. (Fig. 3).

## Discussion

In this investigation, we showed that salient markers of endothelial activation and blood–brain barrier compromise released early after TBI can be detected using label-free MS, without the need for antibody-based enrichment. This serves as a demonstration that proteins of interest may be detectable in “noisy” clinical samples from acute trauma patients with varying degrees of injury severity and body area involvement. Several of the proteins of interest had been previously detected in these samples using alternate methods, which ensured a level of quality control. We observed that plasma proteins in higher concentrations (in the range of 10,000 to 100,000 pg/mL) were readily detectable without significant processing (protein depletion), which will inform future experimental design.

This pilot investigation provides guidance for future application of MS technology to biomarker science in TBI. First, unless the protein of interest is in higher abundance, immuno-depletion of highly abundant proteins, especially albumin, will improve detection rates in trauma patients. One of the challenges of measuring protein biomarker levels in blood is tissue-typing the markers measured. As we observed in this investigation, a number of informative markers in TBI may be non-specific to brain injury, and may be found in other traumatic injury such as isolated body injury.

As a corollary, lack of specificity to brain injury might not disqualify a marker from being informative to post-TBI sequelae.^7,8^ For example, levels of d-dimer are associated with progression of intracranial hemorrhage, and have been used in models to predict progression of intracranial hemorrhage after TBI.^9,10^ Likewise, interleukin (IL)-1-beta production associates with post-traumatic epilepsy risk; higher cerebrospinal fluid (CSF)/serum IL-1β ratios were associated in one study with increased risk for post-traumatic epilepsy (PTE) over time.^11^ Additionally, non-specific inflammatory markers may be associated with long-term outcome after TBI. High sensitivity C-reactive protein measured within 2 weeks of injury was recently shown to be a prognostic biomarker for disability at 6 months.^12^ A future viable strategy might be to identify new markers via global MS, and then follow identification with careful quantification with digital immunofluorescence technologies. In parallel, the source of the marker might be confirmed via extracellular vesicle analysis.^13,14^

Although early in its application to clinical research in trauma, SRM-MS technology is increasingly deployed as a data-driven biomarker discovery strategy in concert with techniques to analyze “big data.” Investigators recently used a shotgun MS approach to identify differential protein expression in patients after acute orthopedic trauma with and without heterotopic ossification (HO).^15^ Another group identified a number of new potential targets specific to TBI in children by performing a global proteomics experiment using hierarchical clustering with s100b as the reference standard.^16^ The advantage of these data-driven approaches is the lack of investigator bias in marker selection; results may be obtained without the need for *a priori* knowledge. Moreover, starting from a data-driven identification approach may illuminate new biological pathways for future investigation. The ability to detect and quantify proteins independent of specific affinity reagents opens the possibility of identifying novel biological processes relevant to prognosis or therapeutic intervention. A team science approach is probably the most effective way to implement MS technology in neurotrauma research, including MS experts, statisticians, and topical experts all working in concert. TBI researchers can most effectively collaborate with proteomic scientists by familiarizing themselves with the strengths and limitations of MS to design the most effective experiments, given the cost and complexity.

Although much of the focus of TBI blood-based biomarker studies has been concentrated on highly brain-specific proteins (glial fibrillary acidic protein) or modestly brain specific (s100 calcium binding protein b, ubiquitin carboxyl-terminal hydrolase isozyme L1, neuron-specific enolase), these well-studied, frequently cited, blood biomarkers exhibit expression profiles that could limit their diagnostic efficacy after TBI. For example, O'Connell and colleagues^2^ showed numerous alternative candidate protein biomarkers expressed in much higher levels than more commonly studied proteins such as ubiquitin carboxyl-terminal hydrolase isozyme L1. Moreover, global proteomics experiments have identified non-brain-specific markers that may be associated with important central nervous system (CNS) events. In a feasibility study, Hergenroeder and associates^17^ aimed to identify biomarkers in immunodepleted serum from patients with severe TBI by first identifying differential protein expression between patients with acute TBI and healthy controls, then carrying forward 31 candidate markers that were significantly altered after injury. Through a series of models, the investigators found serum amyloid A and C-reactive protein were highly associated with injury, whereas retinol binding protein 4 predicted elevations in intracranial pressure (ICP) with reasonably high sensitivity and specificity.^17^ In contrast to our work, this study focused on samples obtained 40–60 h after the antecedent trauma.

We chose to focus on endothelial activation and blood–brain barrier compromise released early after TBI that had been shown in our work,^18^ or in the work of others,^19,20^ to be related to actionable events after acute TBI at an early time-point. Strengths of our investigation include the breadth of age of subjects, injury severity in subjects, number of subjects analyzed, as well as access to plasma so early after injury. By successfully demonstrating feasibility, we hope to lay the groundwork for future studies in biomarker discovery very early after injury. The goal will be to predict actionable events in intensive care such as the progression of intracranial hemorrhage or worsening cerebral edema.

### Limitations

MS is an expensive, labor-intensive technique, which limited the number of patient samples we could analyze (*n* = 44), although the majority of previously published reports have 20 subjects or fewer. We attempted to select a representative sample from the overall FAINT cohort, but our power to detect difference between groups was limited due to the small sample size. This work was not meant to make any definitive statements on group differences, but rather we hoped to obtain preliminary data on variance of the detected markers. Also, targeted MS is often not as sensitive to low-abundance plasma proteins as immunofluorescence techniques, as we saw that not all proteins we attempted to detect were detected despite their known presence in the samples (ANG1, MCP1, TNFA, VEGFD, TGFB1). This leaves open the possibility of missing important informative markers that might fall into the very low abundance range. Although we view this work as an important preparatory step toward future successful global discovery experiments, we did not conduct a global discovery experiment, and thus our observations might not generalize, although the techniques have elements in common. Finally, these results may only be valid for acute injury considering the time-points at which the samples were collected. Therefore, further studies using antibody-free MS may be needed to assess whether this would be applicable for long-term ICU monitoring and prognostication.

## Conclusion

We detected non-specific upregulation of proteins reflecting blood–brain barrier breakdown early after injury in patients with severe injury. MS techniques to detect low-abundance plasma proteins appear to be feasible and may be informative for biomarker discovery in the future.

## Supplementary Material

Supplemental data

## Abbreviations Used

AIS: Abbreviated Injury Severity Score
Ang1: angiopoietin 1
Ang2: angiopoietin 2
cc: Pearson's correlation coefficient
CI: confidence interval
CNS: central nervous system
CSF: cerebrospinal fluid
CT: computed tomography
FAINT: Fever And Inflammation in Neurotrauma
GCS: Glasgow Coma Scale
HO: heterotopic ossification
ICAM1: intercellular adhesion molecule 1
ICP: intracranial pressure
ICU: intensive care unit
IL: interleukin
IQR: interquartile range
ISS: injury severity score
LC: liquid chromatography
MCP1: monocyte chemoattractant protein 1
MMP9: matrix metalloproteinase 9
MS: mass spectrometry
PAI1: plasminogen activator inhibitor-1
PTE: post-traumatic epilepsy
SD: standard deviation
SRM: selective reaction monitoring
TBI: traumatic brain injury
TNFA: tumor necrosis factor alpha
VCAM1: vascular adhesion molecule 1
