# Supplementary material for: Antibody-Free Mass Spectrometry Identification of Vascular Integrity Markers in Major Trauma
Source: Neurotrauma Rep. 2021 Jul 1;2(1):322–9. doi: 10.1089/neur.2021.0007 (PMC8310742; doi:10.1089/neur.2021.0007)
Supplement: Supplemental data [file Supp_TableS1.pdf]

# Supplemental Table 1

| Protein Target | Peptide detected |
|---|---|
| VCAM1 | NTVISVNPSTK |
| ICAM1 | DGTFPLPIGESVTVT**R** |
| MMP9 | AVIDDAFAR |
| PAI1 | GAVDQLTR |
| Ang 2 | ISSISQPGNDFST**K** |

Peptide sequences used to detect proteins of interest.
